# Low Doses of Ochratoxin-A Decrease IgY and IgA Production in Broiler Chicks

**DOI:** 10.3390/toxins10080316

**Published:** 2018-08-06

**Authors:** Shahzad A. Khan, Emerson J. Venancio, Eduardo V. Fernandes, Elisa Y. Hirooka, Alexandre Oba, Karina K. M. C. Flaiban, Eiko N. Itano

**Affiliations:** 1 Department of Pathologic Sciences, State University Londrina, P.O. Box 10.011, Londrina 86057-970, PR, Brazil; emersonj@uel.br (E.J.V.); eduardovignoto@uel.br (E.V.F.); 2 Department of Food Science and Technology, State University Londrina, P.O. Box 10.011, Londrina 86057-970, PR, Brazil; elisahirooka@hotmail.com; 3 Department of Zootechny, State University Londrina, P.O. Box 10.011, Londrina 86057-970, PR, Brazil; oba@uel.br; 4 Department of Preventive Veterinary Medicine, State University Londrina, P.O. Box 10.011, Londrina 86057-970, PR, Brazil; kkflaiban@uel.br

**Keywords:** IgA, IgY, leukocytes, lymphocytes, mycotoxins, toxins

## Abstract

The mycotoxin, ochratoxin-A (OTA), produced by some fungi, and is a natural contaminant of many foods and animal feeds worldwide. Due to its toxic effects, the recommended maximum daily intake of OTA for poultry feeds is 0.1 mg OTA/kg (ECR2006/575/EC); this dose does not induce changes in hepatic/renal parameters, but decreases thymus size and serum globulin concentrations. Accordingly, in this study, we assessed quantitatively the total circulating IgY and IgA serum levels, in chicks consuming a 0.1 mg OTA/kg diet (limit) and higher doses (0.3–1.1 mg OTA/kg diet) for 14 or 21 days. We also evaluated other immunological parameters (thymus, bursa of Fabricius, and spleen weights and leukocyte profiles) at day 21. Decreased IgY serum levels were observed in all OTA-treated groups (*p* < 0.05). In the low-dose group, IgA levels were decreased on day 21, but not on day 14. The size of the thymus and the bursa of Fabricius was decreased in all OTA-treated groups (*p* < 0.05), whereas reduced spleen size and altered leukocyte profiles were detected only in the high-dose group (*p* < 0.05). We concluded that chronic exposure to OTA, even at the recommended highest dose, affected IgY and IgA production in chicks.

## 1. Introduction

Ochratoxin A (OTA) is a critical mycotoxin that causes various harmful effects in animals and humans. This mycotoxin is produced by filamentous fungi, such as *Aspergillus* spp., mainly *A. carbonarius* and *Penicillium* spp. *A. ochraceus* was the first fungus found to produce OTA [1,2].

One of the main routes of exposure is through the consumption of contaminated food/feed, and consumption of this toxin by commercial animals, such as poultry, can have a substantial economic impact [3]. Mycotoxin contamination can occur at any stage in the feed production chain. The occurrence of OTA or OTA toxigenic fungi has been detected in cereals and animal feeds worldwide [4,5,6,7,8,9,10].

One of the essential characteristics of OTA is its thermostability. According to Boudra et al. [11], even at high temperatures (up to 100–250 °C), OTA is not completely eliminated. This characteristic may contribute to its persistence, even in heat-processed food/feed.

Human or animal exposure to OTA has been reported worldwide [12,13,14,15], having substantial effects on public health. OTA is classified by the International Agency for Research on Cancer as a Group 2B agent (possible human carcinogen) [16] and has been shown to have a wide variety of toxicological effects, such as mutagenicity, teratogenicity, nephrotoxicity, neurotoxicity, hepatotoxicity, hematotoxicity, and immunotoxicity, resulting from natural human or animal exposure to OTA or experimental animal exposure to OTA [17,18,19,20].

In the poultry industry, the toxic effects of OTA are most often observed as decreased growth rates [3,6]. Additionally, immunotoxicity, which causes increased susceptibility to infectious diseases, also contributes to economic losses in the poultry industry [3,21]. In poultry, OTA also decreases the sizes of central lymphoid organs, including the thymus and bursa of Fabricius [22,23], which produce T and B cells, respectively [24]. Moreover, the sizes of secondary lymphoid organs, such as the spleen, have also been shown to be decreased in poultry exposed to OTA [22].

Antibodies are vital for defense against various infectious diseases. Three classes of immunoglobulins (IgY, IgA, and IgM) are expressed in chicks [24]. Among these, IgY is most abundant in serum and is critical in the secondary immune response, while IgA plays an essential role in mucosal defense [24] Notably, OTA has been shown to affect the humoral response in chicks by altering the levels of specific antibody titers in response to sheep red blood cells or viruses, such as Newcastle disease virus [25,26,27,28]. However, total immunoglobulins (Ig) quantification is very scarce and is typically performed using immunodiffusion [29], and more sensitive assays are needed to conclusively elucidate the changes in Ig concentrations in response to OTA. In addition, high doses of OTA are often used to assess the effects of OTA on the immune system [23,25,26,27,28,29]. Pozzo et al. [30] used the maximum level of OTA suggested by the European Commission Recommendation 2006/576/EC (0.1 mg OTA/kg) for poultry feeds [31] and observed reductions in thymus weights and in total serum protein, albumin, and alpha-, beta-, and gamma-globulin levels; although other parameters (such as animal performance, hematological parameters, liver enzymes, or renal function parameters) were not affected.

Accordingly, in this study, we evaluated the total circulating IgY and IgA serum levels using ELISAs and other immunological parameters (thymus, bursa of Fabricius, and spleen weights and circulating leucocytes profiles) in chicks consuming 0.1 mg OTA/kg diet (European Commission Recommendation 2006/576/EC) or higher doses for 21 days. Our findings provided important insights into the effects of OTA on the central lymphoid organs and immune responses in chicks even at low level.

## 2. Results

### 2.1. Serum Levels of Total IgY and IgA in Chicks

**Figure 1:**
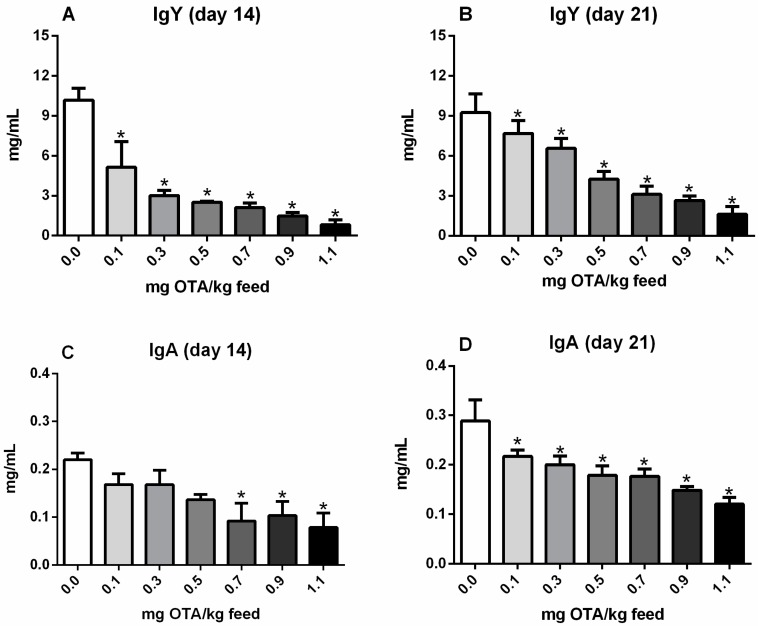
Serum levels of total IgY and IgA in chicks treated with Ochratoxin A (OTA) by capture ELISA. (**A**) IgY on day 14, (**B**) IgY on day 21, (**C**) IgA on day 14, and (**D**) IgA on day 21 in chicks treated with 0.0, 0.1, 0.3, 0.5, 0.7, 0.9, and 1.1 mg OTA/kg feed. *n* = 6 (for each group). * significant reduction compared with the control not exposed to OTA (*p* < 0.05).

The total IgY serum level was decreased in all groups consuming OTA-contaminated feed (0.1, 0.3, 0.5, 0.7, 0.9, and 1.1 mg OTA/kg feed) compared with that in the control group on days 14 (*p* < 0.05; Figure 1A) and 21 (*p* < 0.05; Figure 1B). However, the total IgA serum level was significantly reduced only in groups of chicks treated with 0.7, 0.9, and 1.1 mg OTA/kg feed for 14 days (*p* < 0.05; Figure 1C). On day 21, similar to IgY, IgA levels were significantly decreased in all groups consuming OTA-contaminated feed (0.1, 0.3, 0.5, 0.7, 0.9, and 1.1 mg OTA/kg feed; *p* < 0.05; Figure 1D).

### 2.2. Relative Weights of the Bursa, Thymus, and Spleen

**Figure 2:**
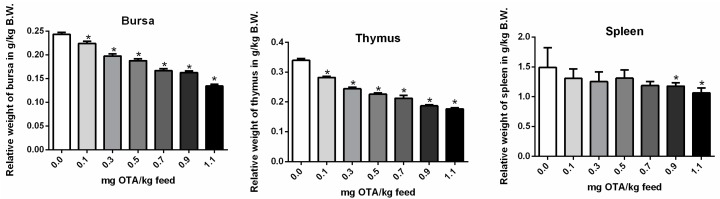
Relative weights of the bursa, the thymus, and the spleen. Relative weights of the bursa (**A**), thymus (**B**), and spleen (**C**) in OTA-treated chicks. *n* = 6 (for each group). (* *p* < 0.05). B.W.: Body Weight.

The relative weights of the bursa and thymus (Figure 2A,B) were significantly decreased in all OTA-treated groups compared with the control group (*p* < 0.05). However, the relative spleen weight was significantly decreased only at higher dose of OTA (0.9 and 1.1 mg OTA/kg feed) treated groups as compared with the control (*p* < 0.05; Figure 2C). No significant differences were observed for all other OTA-treated groups.

### 2.3. Circulating Leukocytes and Differential Counts

**Figure 3:**
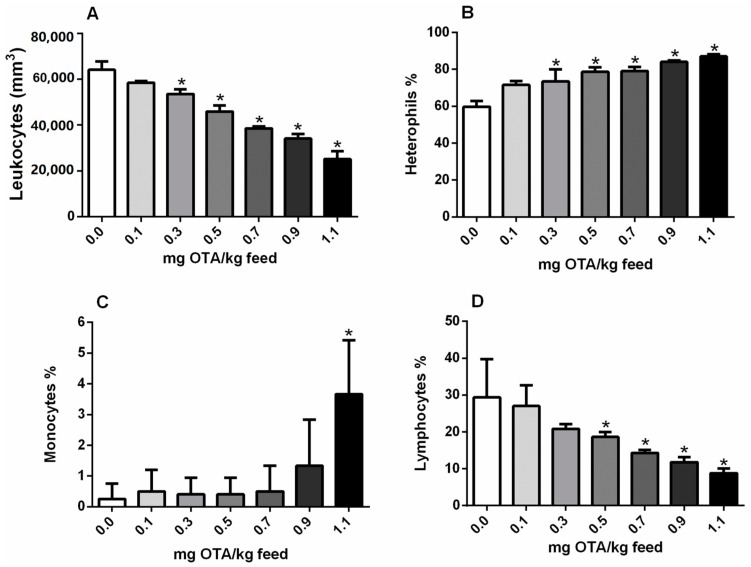
Effects of OTA treatments in broiler chicks on leukocyte numbers. (**A**) Total circulating leukocytes (mm^2^); (**B**) heterophils (%), (**C**) monocytes (%), and (**D**) lymphocytes (%). *n* = 6 (for each group). * Significant compared with the control not exposed to OTA (*p* < 0.05).

No changes were observed in circulating leukocyte concentrations or percentages of lymphocytes, monocytes, or heterophils at a dose of 0.1 mg OTA/kg feed. Total circulating leukocytes were significantly reduced in chicks treated with 0.3, 0.5, 0.7, 0.9, and 1.1 mg OTA/kg feed compared with that in the control group (*p* < 0.05; Figure 3A). Additionally, lymphocytes were significantly reduced in OTA-treated chicks (0.5, 0.7, 0.9, and 1.1 mg OTA/kg feed) compared with that in the control group (*p* < 0.05; Figure 3D). In contrast, the number of monocytes was increased in chicks exposed to 1.1 mg OTA/kg feed (Figure 3C), and the number of heterophils was increased in chicks fed with 0.7 mg OTA/kg or more (Figure 3B) as compared with those in the control group (*p* < 0.05). No differences in eosinophil levels were detected (*p* > 0.05) (data not shown).

### 2.4. Total Serum Protein Concentrations in Chicks Exposed to OTA

**Figure 4:**
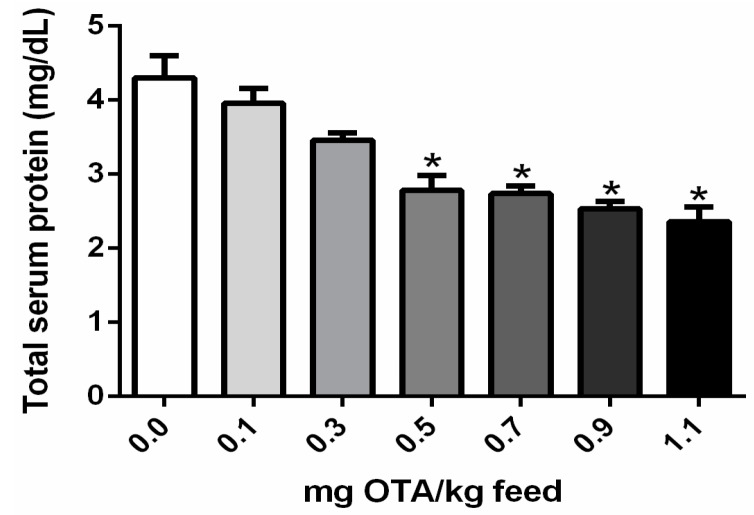
Total serum protein concentrations in chicks exposed to OTA. Protein concentrations are expressed in mg/dL; *n* = 6 (for each group). * Significance threshold, as compared with the control not exposed to OTA (*p* < 0.05).

Total serum protein concentration was significantly reduced in chicks treated with 0.5, 0.7, 0.9, and 1.1 mg OTA/kg feed in comparison with the control group, not exposed to OTA (*p* < 0.05; Figure 4).

## 3. Discussion

The maintenance of a competent immune system is essential for preventing infectious diseases in commercially important animals. The mycotoxin OTA induces immunosuppression, and prolonged exposure to OTA at low doses have more significant effects on the immune system than acute exposure at high doses [22]. In this study, we investigated the effects of OTA at various doses, including the recommended maximum dose (ECR2006/575/EC) via oral exposure. Additionally, because OTA-induced toxicity may have a greater impact during early life, when the immune system is not fully developed and when many vaccines are often administered (younger broiler chicks were used in this study). Our results showed that serum IgY levels were decreased in chicks consuming 0.1 mg OTA/kg feed, whereas IgA suppression occurred only at higher doses (0.3 mg OTA/kg feed), on day 14, which suggested that OTA may have more dramatic effects on IgY rather than on IgA production. However, after a more extended period of OTA exposure (21 days), the levels of circulating IgY and IgA antibodies were decreased, even for low-dose OTA. External secretion of IgA is extremely important for mucosal defense [29]. However, in this study, only blood samples were evaluated, and further studies are required to examine whether IgA secretion may be suppressed.

The decreases in antibody levels induced by OTA may be due to its effects on different stages of the immune response. Several studies have shown that OTA intoxication leads to decreases in the sizes of central or primary lymphoid organs, including the thymus and bursa of Fabricius [22,23]. In this study, we found that the thymus and bursa of Fabricius showed decreased sizes (weights), even after low dose exposure (0.1 mg OTA/kg feed). Pozzo et al. [30] detected reduce thymus sizes, but not bursa of Fabricius sizes, in chicks consuming 0.1 mg OTA/kg feed. These discrepancies may be related to the difference in the broiler chickens used; we used Cobb broiler chickens, whereas Pozzo et al. [30] used Hubbard broiler chickens.

Additionally, secondary lymphoid organs, such as the spleen, are also affected by OTA intoxication, showing decreased sizes [22]. In this study, the reduction in spleen’s size was observed only for chicks consuming 0.9 mg OTA/kg feed, suggesting that this organ may be more resistant to OTA, than the primary lymphoid organs. Further studies are required to confirm these findings.

Decreased antibody levels have been shown to be associated with decreased leukocyte counts induced by OTA intoxication [32,33]. Low leukocyte levels are associated with immunodeficiency in animals [34]. In this study, we found that decreased leukocyte numbers were detected in animals consuming 0.3–1.1 mg OTA/kg feed. These results were consistent with findings reported in other studies with higher OTA exposure levels (0.5–8.0 mg OTA/g feed and 0.75–3 mg OTA/g feed) [35,36].

Reduced leukocyte numbers are associated with decreased circulating lymphocytes [37]; however, this association was observed only with 0.5 mg OTA/kg feed in this study. Thus, the decreased percentage of lymphocytes observed in this study would not be expected to contribute to the decline in total leukocyte numbers detected with 0.3 mg OTA/kg feed, since the reduction in leukocytes was observed with a dosage of 0.3 mg OTA/kg feed. Additionally, the reduced percentage of lymphocytes may have been related to the increased heterophil and monocyte percentages observed in this study, similar to the results of another study [38]. Moreover, similar decreases in lymphocyte percentages with simultaneous increases in heterophil percentages have also been observed by Bartlomiej et al. [39].

Protein production may be inhibited by OTA, thereby resulting in reduced antibody production [28]. Indeed, in this study, we also found decreased levels of serum total protein, although this decrease was detected only with 0.7 mg OTA/kg feed. Therefore, several factors must be involved in the observed decreases in IgY and IgA production. The inhibition of protein synthesis by OTA may be due to its effects on phenylalanine t-RNA synthase and phenylalanine hydroxylase. Moreover, OTA acts as a regulator of intracellular transcription of many proteins [40].

Modulation of cytokine levels and decreases in cellular immune responses associated with OTA intoxication have also been demonstrated [21,41]. Owing to the complexities of the immune response, additional studies of the effects of OTA on various stages of the immune response, including both innate and adaptive immunity, are required.

In this study, we did not measure OTA concentrations in blood or tissue samples in exposed animals. However, the observed effects were more evident in groups that received increased doses of OTA in contaminated feed. Therefore, further studies are needed to confirm the differences in OTA concentrations in blood and tissues.

Overall, our results showed that even low doses of OTA (recommended maximum limit), consumed in feeds over several weeks, induced suppression of systemic IgY and IgA production and decreased thymus and bursa of Fabricius weights in broiler chickens. These results suggested that it is necessary to evaluate even smaller doses for long-term repetitive exposures to confirm the maximum dose that does not affect the immune system, which seems to be highly susceptible to OTA.

## 4. Conclusions

We concluded that even at the maximum recommended dose of contamination (0.1 mg OTA/kg feed, maximum limit in poultry feed by European Commission Recommendation 2006/576/EC), repetitive exposures affected the production of both IgY and IgA in broiler chicks exposed to OTA.

## 5. Materials and Methods

### 5.1. Experimental Chicks and Their Management

This study was carried out with 42 mash, 1-day-old, specific pathogen-free broiler chicks (Cobb) from a local hatchery in Londrina, Parana, Brazil, for 21 days. All chicks used in the present study were from the same breeding flock. The experimental rooms and sheds were thoroughly cleaned and subsequently fumigated with KMnO_4_ and formalin (1:2) before housing. The chicks were kept under strict hygienic conditions and maintained on broiler mash from day 1 until the end of the experiment. Feed [42] and water were given ad libitum, and no probiotics, antibiotic growth promoters, or therapeutic drugs were administered during the entire experimental period.

### 5.2. Preparation of OTA-Contaminated Feed

OTA (CAYM 11439; Cayman Chemical Company, Ann Arbor, MI, USA) was resuspended in ethanol (1 mg OTA/10 mL). This suspension was then evenly mixed in the required quantity of basal feed to prepare the experimental feed containing the desired concentration of OTA three days before the commencement of the experiment to ensure uniform distribution.

### 5.3. Experimental Design

On day 1, chicks were divided into seven groups, with six chicks in each group. The control group was provided a feed that was not contaminated, whereas the experimental groups were provided an OTA-contaminated feed (0.1, 0.3, 0.5, 0.7, 0.9, and 1.1 mg OTA/kg feed) for up to 21 days. Animal rooms were kept at ~33 °C for the first week, at ~32 °C for the second week (newborn chicks present sensitivity to cold), and ~24 °C for the remainder of the study. The rooms were also maintained at 60% relative humidity and with a 12-h/12-h light-dark cycle. All chicks had access to fresh water and feed ad libitum for 21 days. All animal experiments were conducted according to the rules and regulations of the Animal Care and Ethics Committee and carried out after the approval by the Ethics Committee on the Use of Animals, State University of Londrina, PR, Brazil (CEUA 18419.2013.89) Date of approval 29 November 2013.

### 5.4. Quantification of IgY and IgA

On day 14 and 21 after the beginning of OTA treatment, 2 mL blood was collected from the wing vein. After coagulation, serum samples were obtained by centrifugation and were stored at (−20 °C) until use. Immunocapture ELISA was performed for determination of IgY and IgA levels in the serum of all control and experimental groups using commercial kits (Bethyl Laboratories, Montgomery, TX, USA). Serum was diluted at 1:200,000 for IgY and 1:500 for IgA. Concentrations of IgY and IgA were calculated based on standard curves.

### 5.5. Relative Weights of the Bursa, Thymus, and Spleen

On day 21, all the chicks from each group were slaughtered by the half neck method. The bursa of Fabricius, the thymus, and the spleen were weighed separately, and the respective weights relative to the total body weights were calculated.

### 5.6. Circulating Leukocyte Profiles and Differential Counts

On day 21, prior to slaughtering, 2 mL blood was collected from the wing vein of each bird into 5% ethylenediaminetetraacetic acid for hematological analysis. Leukocytes, lymphocytes, heterophils, eosinophils, and monocytes were quantified using an autoanalyzer (Beckman Model 700 Analyser, Woerden, The Netherlands).

### 5.7. Total Serum Protein Concentrations in Chicks Exposed to OTA

Serum samples collected from birds in each group at the end of the experiment were used to determine concentrations of serum total protein. The measurements were carried out with a spectrophotometer using commercially available kits (Diasys Diagnostic system GmbH, Holzheim, Germany).

### 5.8. Statistical Analysis

Before statistical analysis, all data were evaluated for homogeneity (Levin’s tests) and normality (Kolmogorov tests). All data were subjected to one-way analysis of variance. Means of different groups were compared by Bonferroni tests using GraphPad Prism statistical package 5.01. Data were considered significantly different when *p* values were less than 0.05.
